# Antibiotics Resistance Profile of Clinical Isolates of *Pseudomonas aeruginosa* Obtained from Farwaniya Hospital in Kuwait Using Phenotypic and Molecular Methods

**DOI:** 10.3390/antibiotics14060539

**Published:** 2025-05-24

**Authors:** Rawan Saad Althaferi, Wadha Ahmed Alfouzan, Abu Salim Mustafa

**Affiliations:** 1 Department of Microbiology, Faculty of Medicine, Kuwait University, Kuwait City 13110, Kuwait; rawan.althafiri@grad.ku.edu.kw (R.S.A.); abu.mustafa@ku.edu.kw (A.S.M.); 2 Microbiology Unit, Department of Laboratory Medicine, Farwaniya Hospital, Ministry of Health, Farwaniya 80000, Kuwait

**Keywords:** *Pseudomonas aeruginosa*, antibiotic resistance, MDR, resistance genes, mutations

## Abstract

**Background/Objectives:** The World Health Organization has recognized *Pseudomonas aeruginosa* as a multidrug-resistant bacterium that presents public health concerns. This study aimed to evaluate the prevalence of MDR *P. aeruginosa* isolates along with their susceptibility profiles and determine the genetic basis of resistance. **Methods:** A total of 100 *P. aeruginosa* isolates were cultured on MacConkey agar with various specimens from patients admitted to ICUs and wards. Species identification was performed for each isolate using the VITEK^®^ 2 system. Each isolate was tested for susceptibility to specific antibiotics by the broth microdilution method. The resistance genes were detected by molecular methods, i.e., PCR and Sangar sequencing. **Results:** Among the 100 *P. aeruginosa* isolates tested phenotypically, 33 MDR *P. aeruginosa* isolates were detected. The aminoglycoside group of antibiotics showed the least resistance against *P. aeruginosa*, with increasing resistance to carbapenems and ciprofloxacin. The most prevalent detected genes responsible for resistance were *blaVEB*, *blaVIM*, *aac (6′)-Ib*, and *qnr S*. DNA sequencing results for the MDR isolates showed that 14 isolates had Thr-83> Ile mutation in *gyrA*, and 12 isolates had Ser-87>Leu mutation in *parC* genes. **Conclusions:** We conclude that the low rates of resistance to certain antibiotics, such as amikacin and piperacillin-tazobactam, seem encouraging to be effective for the treatment of *Pseudomonas* infections. Furthermore, the prominent mechanisms of resistance to fluoroquinolones in clinical strains of *P. aeruginosa* include mutations in *gyrA* and *parC* genes. These findings highlight the necessity of molecular diagnostics in guiding therapy and the potential need for broader surveillance.

## 1. Introduction

*Pseudomonas aeruginosa* is a rod-shaped and Gram-negative bacterium that can cause a wide range of infections in individuals with both normal and compromised immune systems. Treating the infections caused by this organism in the context of modern medicine poses significant challenges, mostly attributable to its propensity to infect individuals with impaired immune systems, its remarkable adaptability, resistance to antibiotics, and a diverse array of dynamic defense mechanisms. This microorganism is commonly characterized as an opportunistic pathogen, contributing to the development of nosocomial infections, e.g., ventilator-associated pneumonia and catheter-associated urinary tract infections, etc. [1].

Prior research has indicated that patients in intensive care units (ICUs) play a significant role in the generation, transmission, and enhancement of drug-resistant organisms [2]. This is mostly due to the enhanced selection pressure that results from treating these patients with antibiotics, which leads to the emergence of drug-resistant bacteria [2].

The emergence of antibiotic resistance worldwide has become a significant barrier to the efficacy of available antibiotics. The Centers for Disease Control and Prevention (CDC) has identified antimicrobial resistance (AMR) as an urgent global public health concern. It has been estimated that, in 2019, AMR was associated with about 5 million deaths, and it was solely responsible for at least 1.27 million deaths worldwide [3]. Additionally, the World Health Organization (WHO) has recognized *P. aeruginosa* as a type of multidrug-resistant (MDR) bacterium that presents a specific risk in healthcare settings, including hospitals, nursing homes, and among patients who utilize medical devices such as ventilators and blood catheters [4]. The efficacy of a large number of antibiotics, including carbapenems and third-generation cephalosporins, in treating infections caused by MDR bacteria has decreased significantly [4].

The emergence of carbapenem resistance in strains of *P. aeruginosa* includes carbapenemases driven by plasmids or integrons, overexpression of efflux systems, diminished expression of porins, and increased action of chromosomal cephalosporins [5]. The primary mechanism underlying resistance is mostly attributed to the loss of OprD, which causes resistance to imipenem and meropenem [5]. Furthermore, the multidrug efflux systems that are responsible for the development of resistance to quinolones, chloramphenicol, and various other antimicrobial agents also contribute to the resistance against carbapenems [5].

*P. aeruginosa* exhibits resistance to three primary classes of antibiotics, namely β-lactams, aminoglycosides, and fluoroquinolones. The major mechanisms by which β-lactam resistance is acquired by mutation involve alterations to the penicillin-binding protein (PBP3) target protein, reduced antibiotic absorption, enhanced export, and degradation of the antibiotic molecule. Moreover, the acquisition of antibiotic-degrading enzymes, specifically β-lactamases, from other bacterial species is facilitated by the process of horizontal gene transfer [6]. Resistance can potentially be impacted by alterations in metabolism and increased development of biofilms [7]. *P. aeruginosa* exhibits inherent susceptibility to ceftazidime, aztreonam, and carboxypenicillins; nevertheless, it has the potential to acquire resistance by a genetic mutation leading to overexpression of AmpC beta-lactamase. *P. aeruginosa* produces a molecular class C inducible AmpC beta-lactamase, which is also known as a cephalosporinase [7].

The aminoglycoside class of antibiotics, including gentamicin, tobramycin, and amikacin, exert their inhibitory effects on bacterial protein synthesis through their interaction with ribosomal 30S subunits [8]. The development of resistance to aminoglycosides in *Pseudomonas* is facilitated by the presence of transferable aminoglycoside modifying enzymes (AMEs), diminished permeability of the outer membrane, active efflux mechanisms, and, in rare cases, modifications to the drug’s target [8]. In strains that exhibit resistance, aminoglycosides undergo modifications by the action of enzymes such as aminoglycoside, phosphotransferases (APH), aminoglycoside acetyltransferase (AAC), or aminoglycoside nucleotidyltransferase (ANT), which respectively introduce a phosphate group, an acetyl group, or an adenylate group This process leads to the formation of modified antibiotics that exhibit a reduced binding affinity to their target, the 30S ribosomal subunit within the bacterial cell [8]. Furthermore, efflux pumps, belonging to the resistance-nodulation-division (RND) family, facilitate the expulsion of drugs and other substances from the bacterial cell. In addition, it has been observed that the MexXY-OprM efflux pump is frequently upregulated, leading to the prevalence of efflux-mediated aminoglycoside resistance [8].

The fluoroquinolones inhibit two essential enzymes, which are DNA gyrase and topoisomerase IV. These enzymes are type II topoisomerases in bacteria with crucial roles in DNA replication [9]. The motif located in the quinolone-resistant determinative region (QRDR) of the *gyrA*/*gyrB* genes, which corresponds to the active site of the enzyme, is a potential location for the occurrence of a mutation in topoisomerase IV [10]. Consequently, the amino acid sequences of both the A and B subunits undergo alterations, leading to a modified topoisomerase II that exhibits a diminished affinity to bind quinolone molecules. Furthermore, it has been observed that the upregulation of efflux mechanisms plays a role by which *Pseudomonas* acquires resistance to fluoroquinolones [10].

In this study, we have evaluated the prevalence of multidrug-resistant (MDR) *P. aeruginosa* isolated from clinical samples obtained from patients admitted to Farwaniya Hospital in Kuwait along with their susceptibility profile and determined the basis of resistance in MDR *P. aeruginosa* isolates by detecting the resistance-causing genes by using molecular methods, i.e., polymerase chain reaction (PCR) and partial gene sequencing.

## 2. Results

### 2.1. Sample Collection

A total of 218 clinical samples Respiratory (n = 144), urine (n = 25), blood (n = 7), and others including wound samples (n = 42) during the study period. The various pathogenic bacterial species isolated from these samples are shown in Table 1. Respiratory samples were the most common source of isolates, indicating the clinical prevalence of ventilator-associated pneumonia and other hospital-acquired respiratory infections [11]. Table 2 presents further information on the bacterial species isolated from samples obtained from wards and ICUs, which is critical for understanding the hospital epidemiology of *P. aeruginosa* and other important pathogens.

### 2.2. Isolation and Identification

**Figure 1:**
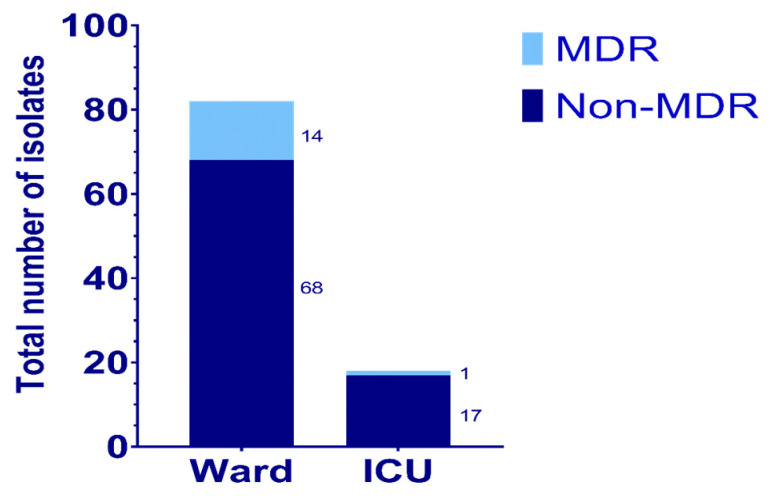
The distribution of *P. aeruginosa* isolated from specimens obtained from the Wards and ICUs in Farwaniya Hospital.

A total of 108 *P. aeruginosa* isolates were obtained from the 218 clinical samples by culturing the specimens on plates containing MacConkey agar (Table 2). These isolates were frozen at −70 °C in a medium that contained glycerol, distilled water, and Brain Heart infusion broth to maintain their viability for subsequent analysis. On re-culturing the isolates from the frozen stocks to perform further experiments, 82 isolates were grown from samples obtained from the wards, and 18 isolates were grown from the samples obtained from the ICUs (Figure 1). The remaining eight isolates failed to grow on re-culturing from the frozen stocks. This could be due to multiple freeze–thaw cycles or loss of viability during long-term storage, a limitation observed in prior research with Gram-negative pathogens [12].

### 2.3. Susceptibility Test by Broth Microdilution Method

The broth microdilution method was used to test the susceptibility of all *P. aeruginosa* isolates obtained from the wards (n = 82) and ICUs (n = 18). The number of isolates resistant to various antibiotics among the wards and ICU isolates is shown in Table 3. The results showed that 75 (91.4%) out of 82 isolates were resistant to at least one antibiotic, and 30 (36.5%) of them were multidrug resistant. This high MDR prevalence most likely reflects increased antibiotic exposure in general wards and may be influenced by prolonged hospitalization and insufficient infection control practices. These factors were previously associated with the emergence of resistance in nosocomial bacteria [13].

Out of the 18 ICU isolates, 4 isolates revealed resistance to at least one antibiotic, and only three isolates were multidrug-resistant (Table 3). The relatively reduced resistance seen in this group could be related to the smaller sample size or more active infection control measures in critical care units. The sensitivity/resistance profile for each sample is shown in Supplementary Table S2.

### 2.4. Molecular Detection of Resistance Genes

Out of 100 isolates of *P. aeruginosa*, 33 isolates were MDR, of which 30 (90.9%) were from the wards and 3 (9%) isolates were from the ICUs. Among 33 *P. aeruginosa* MDR isolates, only 15 isolates were randomly selected to be investigated for the existence of resistance genes. ESBL resistance genes were detected in 3 (20%) that carried the gene *blaVEB*, and 1 (6.6%) carried the gene *blaOXA-10.* In contrast, none of the isolates carried the genes *blaTEM* and *blaSHV*, indicating that these classical ESBL genes were not prevalent in this setting.

Carbapenem resistance genes were detected in 5 (33.3%) isolates carried the gene *blaVIM*, 4 (26.6%) isolates carried the gene *blaNDM*, and 1 (6.6%) isolate carried the gene *blaIMP*. However, the genes *blaOXA-48* and *blaOXA-23* were not detected in any of the MDR isolates, suggesting that metallo-β-lactamases may play a more dominant role in carbapenem resistance in this population, as previously reported in the Gulf region [14]. Aminoglycosides resistance genes were detected in 3 (20%) isolates that carried the gene *aac (6′)-Ib*, 1 (6.6%) isolate carried the gene *ant(3″)-Ia*, and 1 (6.6%) isolate carried the gene *aph(3′)-Ib.* The genes *ant (2″)-Ia*, and *aac(3)-Ia* were not detected in any of the tested isolates. These findings indicate that acetylation and phosphorylation are the most prevalent resistance mechanisms in the current isolate group [8]. The presence of fluoroquinolone resistance genes was detected only in 12 (80%) isolates that carried the gene *qnr S*. The other fluoroquinolone resistance genes were not detected. The dominant presence of *qnrS* is consistent with previous investigations, which have linked this gene to plasmid-mediated quinolone resistance in *P. aeruginosa* [15].

### 2.5. Sequencing of gyrA and parC Genes in MDR Isolates of P. aeruginosa

Mutations were detected in both *gyrA* and *parC* genes, which are key components of the quinolone resistance-determining region (QRDR) in MDR *P. aeruginosa* isolates using DNA sequencing. Out of the 15 MDR isolates investigated, 14 (93.3%) isolates exhibited mutations in the *gyrA* gene in which a point mutation caused alteration from amino acid threonine to isoleucine at codon 83 (Thr>Ile, ACC83ATC) (Table 4). This particular mutation is a well-studied mechanism related to lower fluoroquinolone binding affinity to DNA gyrase, resulting in resistance [10]. Furthermore, a mutation in the *parC* gene in 12 (80%) isolates changed serine to leucine at codon 87 (Ser>Leu, TCG87TTG) (Table 4). This mutation particularly affects topoisomerase IV, which contributes to fluoroquinolone resistance. The high frequency of these double mutations is consistent with global investigations that have identified Thr83Ile and Ser87Leu as the most common QRDR changes in *P. aeruginosa* resistant to ciprofloxacin and other fluoroquinolones [10,16]. Other silent mutations were also detected in MDR *P. aeruginosa* isolates, as shown in Table 4. These findings emphasize the importance of target-site mutations in fluoroquinolone resistance and the need for continuing genetic surveillance of QRDR changes, especially in clinical settings with large antibiotic use.

## 3. Discussion

The present study was designed for a better understanding of the prevalence of multidrug-resistant (MDR) *P. aeruginosa* along with their susceptibility profile and the molecular aspects of the basis of resistance in MDR *P. aeruginosa* in one of the largest general hospitals in Kuwait. In the present study, 100 clinical *P. aeruginosa* isolates were tested. These isolates were obtained from respiratory (42.5%), urine (20.3%), blood (3.7%), and other (33.3%) samples. In agreement with our results, an earlier report from Saudi Arabia isolated *P. aeruginosa* strains primarily from respiratory (42.7%) and urine (22.2%) samples [17]. However, a retrospective study from Kuwait has shown that most of the *P. aeruginosa* isolates were cultured from urine specimens (66.0%), followed by respiratory specimens (13.5%), wound, bone, or other tissue (10.7%), blood (8.6%), and body fluids (1.2%) [18].

The prevalence of MDR *P. aeruginosa* is rising in several regions of the world, posing significant treatment challenges. According to the criteria used to identify MDR as resistant to at least one agent in three or more classes of antibiotics, the rate of MDR *P. aeruginosa* in our study was 33% (wards 90.9%, ICU 9%), which is significant. An earlier study conducted in Kuwait used the same definition for MDR and reported high rates of MDR *P. aeruginosa* isolated from wards (medical 27.2%, surgical 24.7%, pediatric 47.0%, and ICUs (26.3%) [18]. In contrast, a previous review used the same definition and reported 8.1% MDR from Qatar and 7.3% MDR *P. aeruginosa* from Saudi Arabia [13]. The high MDR rate detected emphasizes the critical necessity for routine susceptibility testing and local antimicrobial stewardship policies. Thus, periodic evaluations of the MDR trend among *P. aeruginosa* isolates will be required to facilitate drug resistance pattern monitoring at medical facilities in Kuwait.

Considering the excessive use of antibiotics in healthcare facilities and the continuing spread of antibiotic resistance, the evaluation of resistant isolates by susceptibility testing appears to be crucial to prevent the emergence of new resistant strains. The development of carbapenem resistance in strains of *P. aeruginosa* involves a wide range of conditions. Contributing components that have been found include carbapenemases such as metallo-beta-lactamases IMP, NDM, and VIM. In addition, the Class D, OXA-48 enzyme driven by plasmids or integrons, increased expression of efflux systems, diminished expression of porins, and increased action of chromosomal cephalosporins [19]. In the present study, the most common *P. aeruginosa* phenotypes were resistant against carbapenem antibiotics, including imipenem and meropenem, with 30% and 28%, respectively. This high resistance to carbapenems suggests the improper use of broad-spectrum antibiotics and probably the existence of carbapenemase-producing bacteria.

Compared to a previous study, our result was much higher than a previous study from Nepal, which revealed 10.29% imipenem resistance in *P. aeruginosa* isolates [20]. However, the findings in the present study are consistent with those of another study, which used 233 clinical isolates of *P. aeruginosa* from a tertiary hospital in Saudi Arabia. This study showed significantly high resistance to meropenem and imipenem at rates of 50% and 79.6%, respectively [21]. Resistance may be caused by the production of metallo-β-lactamases (MBL), which can be either encoded by genes present in chromosomes or plasmids. Carbapenem hydrolyzing enzymes can be classified as class B-metallo β-lactamases, class D-oxacillinases, or class A-clavulanic acid inhibitory enzymes [22]. In addition to drug susceptibility testing using phenotypic methods, this study has also determined the prevalence of antimicrobial resistance genes in *P. aeruginosa* isolates using PCR and Sangar sequencing. In our study, the genotypic method detected *bla*_VIM_ as the most prevalent gene in carbapenem-resistant *P. aeruginosa* (CRPA) (33.3%), followed by *bla*_NDM_ (26.6%). *bla*_VIM_ and *bla*_NDM_ are shown to be the most prevalent carbapenemase genes in the majority of the Arabian Peninsula [14,23]. However, in other studies, *blaOXA-48* was the most prevalent gene in carbapenem-resistant *P. aeruginosa* (46.88% and 37.43% isolates), followed by *bla*_VIM_ gene (31.25% isolates), and *bla*_NDM_ gene (37. 5%, and 5.03% isolates) respectively [24,25]. In addition to carbapenemase genes, the results showed that the prevalent gene for ESBL production was *bla*_VEB,_ which was detected in 3/15 (20%) MDR isolates. It has been observed that the presence of ESBL genes in *P. aeruginosa* isolates varies based on geographical locations [26]. The findings from Iran (93.02%) corroborated our results on the prevalent gene, *bla*_VEB_ [27]. In contrast, another study reported the prevalent gene to be *bla*_PER-1_ [28]. Although no *bla*_CTX_, *bla*_TEM_, and *bla*_SHV_ were detected in this study, MDR *P. aeruginosa* encoding these genes has been reported in various neighboring countries, i.e., Sudan, Iran, etc. [29,30].

OXA-10 is one of the OXA β-lactamases group, which originally had a hydrolytic effect on both oxacillin and cloxacillin antibiotics [31]. Generally, this group does not affect extended-spectrum β-lactams significantly. However, OXA-10 can hydrolyze ceftriaxone, cefotaxime, and aztreonam. To our knowledge, the present study has, for the first time, reported the detection of OXA-10 in 6.6% of MDR isolates from Kuwait. These rates are considered low compared to other geographical areas, such as Egypt, where OXA-10 was detected at a rate of 33.3%, and Iran at a rate of 23.6% [31,32].

Another important finding in this study is the low resistance rate of *P. aeruginosa* isolates (7%) to piperacillin-tazobactam, followed by aminoglycosides (AMI 15%, GN 20%, TOB 18%), which are the backbones for the treatment of infections caused by *Pseudomonas*. Similar results have been reported in a study examined clinical isolates of *P. aeruginosa* from major hospitals throughout Saudi Arabia’s seven administrative areas, revealing that the aminoglycoside class exhibited the highest susceptibility ranging from 57.3 to 76.8% in comparison to β-lactams, fluoroquinolones, and polymyxins. The most significant susceptibility rate was observed for amikacin at 76.8% [33]. However, our finding is contrary to previous studies from India that suggested a significantly higher rate of resistance to aminoglycoside antibiotics, ranging from 50% to 67% [21].

Some organisms have developed enzymes that inactivate amikacin as well, even though it was developed to be a poor substrate for the enzymes that cause inactivation by phosphorylation, adenylation, or acetylation [9]. Amikacin appears to be an effective treatment for infections caused by *Pseudomonas*. Therefore, to prevent the rapid development of resistance strains, the use of amikacin should be limited to severe nosocomial infections. *P. aeruginosa* may deactivate aminoglycosides by utilizing aminoglycoside-modifying enzymes (AMEs), which have varying activity against different aminoglycosides. For instance, AAC(6′)-Ia inactivates amikacin, but AAC(6′)-Ib’ inactivates gentamicin and tobramycin [34]. The present research investigated the prevalence of five major aminoglycoside-resistant genes, including *aac(6′)-Ib* in *P. aeruginosa*. In total, the *aac (6′)-Ib* (20%) gene was the most prevalent AME gene among all the aminoglycoside-resistant *P. aeruginosa* followed by *aph (3″)-Ib* (6.6%), and *ant (3″)-Ia (6.6%).* This highlights the importance of the *aac(6′)-Ib* gene as a significant contributor to tobramycin and amikacin resistance in clinical isolates of *P. aeruginosa*. The reports from Iran have shown that the *aac (6′)-II* gene was the most prevalent AME gene detected in MDR *P. aeruginosa* [35,36]. However, the prevalence of *ant (3″)-Ia* in Iran was higher (18.3%) than our findings [36].

Two mechanisms mediate fluoroquinolone resistance. The first is mutations in the genes that encode the quinolone targets DNA gyrase and topoisomerase IV enzymes. The second mechanism is the genes responsible for plasmid-mediated quinolone resistance, including quinolone resistance (*qnr*) genes encoding proteins that block quinolones by target modification [37]. In our investigation, the ciprofloxacin resistance rate was 28%. However, earlier studies from Kuwait and other regions of the world have concluded a significantly higher rate. In Kuwait, resistance to ciprofloxacin was 75% and 28%, respectively [38,39]. Similar rates were also reported from southwest Iran (59.4%) and Riyadh (55.5%) [21,40]. In contrast, ciprofloxacin resistance has been reported at a lower level (16.5%) in Makkah [20]. The variation in ciprofloxacin resistance across different studies is suggested to be associated with the frequency of fluoroquinolone consumption and the availability of oral doses [41]. This study also attempted to detect five genes responsible for quinolone resistance (*qnrA*, *qnrB*, *qnrC*, *qnrD*, and *qnrS*) in *P. aeruginosa* isolates resistant to quinolones. As per our results, *qnrS* was detected in 86.6% of the isolates, but none of the other *qnr* genes were detected. Like our study, several studies from the Arabian Peninsula have demonstrated that the *qnrS* was the primary gene mediating quinolone resistance among MDR *P. aeruginosa* isolates [42,43,44]. In contrast to the results of these studies, a study from Iran found that *qnrB* and *qnrA* genes were the predominant quinolone-resistant genes at frequencies of 29.2% and 25.8%, respectively [40].

The use of molecular methods to investigate the presence of resistance genes in our study confirms that the molecular mechanisms were responsible for phenotypic resistance and highlights the function of plasmid-mediated resistance, which presents questions regarding horizontal gene transfer. Furthermore, it supports the use of routine molecular diagnostics, particularly for surveillance and outbreak control.

The fluoroquinolone class of drugs, including ciprofloxacin and levofloxacin, are crucial for treating *P. aeruginosa* infections. However, *P. aeruginosa* frequently develops resistance to these drugs following antibiotic therapy. One of the primary mechanisms by which fluoroquinolone resistance develops is the mutation in the genes encoding gyrase and topoisomerase IV. The results of this study show that 93.3% of the resistant isolates had mutations in *gyrA*, and 100% had mutations in *parC*. The most frequent mutation in *gyrA* was the conversion of threonine (a polar amino acid) to a non-polar isoleucine at codon 83, and one of the frequent mutations in *parC* changed serine to leucine at codon 87. 

Another recent study has reported these amino acid changes in *gyrA* and *parC* in 100% and 19% of the ciprofloxacin-resistant *P. aeruginosa* isolates, respectively [16]. However, the MIC values of the tested fluoroquinolone resistance isolates in these studies were much higher compared to our MIC values. Additionally, another study indicated that 28% of the examined isolates showed resistance to both tested fluoroquinolones. All fluoroquinolone-resistant isolates had the same single mutation in gyrA (Thr-83-Ile), whereas 20% possessed a single mutation in parC (Ser-87-Leu) [16]. However, other point mutations previously reported, such as Asp-87 → Asn, Thr-132 → Met, were not detected in our isolates [16,45]. In this study, among 15 resistant isolates, 1 isolate (isolate no. 123), with a low-level resistance, i.e., MIC = 2 μg/mL, had only a silent mutation in *parC*. Hence, it can be concluded that other resistance mechanisms except for the above-stated point mutations in *gyrA* and *parC* may be responsible for this low-level resistance. Moreover, differences in the MICs of sensitive and resistant ciprofloxacin isolates, which had silent alterations in QRDR of *gyrA* or *parC*, suggest that other resistant explanations may contribute to fluoroquinolone resistance. Fluoroquinolone resistance in *P. aeruginosa* has been associated with efflux pump overexpression (MexAB-OprM, MexCD-OprJ, MexEF-OprN, and MexXY-OprM) as well as mutations in gyrB and parE [46]. Hence, the influence of these mechanisms/genes on ciprofloxacin resistance may be clarified by further studies utilizing whole genome sequencing of our isolates.

## 4. Materials and Methods

### 4.1. Sample Collection

A total of 280 samples from various clinical sites, including respiratory samples, urine, blood, and wound samples, were obtained from adult patients admitted to intensive care units and wards in Farwaniya Hospital from November 2022 until February 2023. The samples were transported to the Microbiology Laboratory at the College of Medicine, Kuwait University, by using transport media. A different number was given to each sample. Exclusion criteria involved only the unlabeled sample. Ethical approval for the study was obtained from the Assistant Undersecretary for planning and quality at the Ministry of Health, Kuwait (Research number 2022/2002).

### 4.2. Isolation and Identification

The samples were cultured on plates containing MacConkey agar and MacConkey agar supplemented with meropenem (1 μg/mL) to isolate *P. aeruginosa*. For an entire day, the plates were incubated at 37 °C. The VITEK^®^ 2 system (bioMérieux, MarcyL’Eʁtoile, France) was used to run bacterial suspensions with turbidity equivalent to 0.5 McFarland standard of each isolate to carry out identification and sensitivity testing. Identification was performed using a VITEK 2 GN card, and susceptibility testing was performed using an AST-419 card (bioMérieux, MarcyL’Eʁtoile, France). The isolates were preserved frozen at −70 °C in a medium that contained glycerol, distilled water, and Brain Heart infusion broth.

### 4.3. Antibiotic Susceptibility Testing by Broth Microdilution Method

Antibiotic susceptibility testing was performed for each isolate processed in the VITEK^®^ 2 system, resulting in any of the following terms: ESBL, aminoglycosides resistant, and fluoroquinolones resistant by using broth microdilution panels, according to Clinical and Laboratory Standards Institute (CLSI) standards. In the current study, seventeen antimicrobial agents were tested for susceptibility tests. The antibiotics included in the broth microdilution panel are meropenem (MERO), gentamicin (GEN), ciprofloxacin (CIP), amoxicillin-clavulanic acid (AUG), Colistin (COL), Tigecycline (TGC), ceftazidime (TAZ), imipenem (IMI), Aztreonam (AZT), ceftolozane-tazobactam (C/T), trimethoprim-sulfamethoxazole (SXT) piperacillin-tazobactam(P/T4), cefotaxime (FOT), ceftazidime-avibactam (CZA), ertapenem (ETP), amikacin (AMI), and tobramycin (TOB).

### 4.4. Molecular Detection of Resistance Genes

Genomic DNA was isolated from the cultures of *P. aeruginosa* according to the method described previously [47]. The resistance genes for different antibiotic groups were detected by polymerase chain reaction (PCR) using gene-specific primers detailed in Supplementary Table S1. The target sequences were amplified using the Gene Amp PCR System 9700 (ThermoFisher Scientific, Waltham, MA, USA), as described previously [47]. In brief, each PCR reaction mixture (25 μL) contained genomic DNA (2 μL) from individual isolates, gene-specific forward, and reverse primers (1.5 μL each), ready to load HotStarTaq^®^ Master Mix (12.5 μL) (QIAGEN, Hilden, Germany), and nuclease-free water (7.5 μL). The PCR protocols and the target genes for each group of antibiotics are given in Table 5.

### 4.5. Sequencing of Resistance Genes

The mutations were detected in *gyrA* and *parC* using Sanger sequencing, as described previously [47,52,53]. In brief, the genomic DNA obtained from the bacterial isolates was subjected to amplification by PCR using the gene-specific primers specified in Supplementary Table S1. The PCR products were purified by using an ExoSAP-IT™ purification kit (Thermo Fisher Scientific) according to the manufacturer’s instructions. The purified PCR product and Thermo Fisher Scientific’s BigDye Terminator v1.1 Cycle Sequencing kit (Waltham, MA, USA) were used to perform the sequencing PCR in a 96-well plate. The sequencing plate was securely fixed within an adapter and subsequently placed within the ABI 3130 Genetic Analyzer (Thermo Fisher Scientific) to determine the sequence of nucleotide bases. The plate manager ID sheet was used to record the sample information, analysis protocol results group, and instrument protocol. The sample plate was placed onto one of the decks, and the plate run ID was associated with the plate. The sequencing run was initiated.

The sequence analysis was conducted using the software provided by the Genetic Analyzer. The identification of homologous sequences and the determination of sequence variations were conducted using the BLASTN tool available on the website of the U.S. National Center for Biotechnology Information (NCBI) (http://www.ncbi.nlm.nih.gov/BLAST/ (accessed on 28 July 2024).

## 5. Conclusions

The present study reveals low to moderate rates of antibiotic resistance in *P. aeruginosa* isolates from a general hospital in Kuwait. The most significant level of antibiotic resistance was observed against carbapenems. An analysis of this resistance trend indicated possible overuse of broad-spectrum antibiotics. Nevertheless, the low rates of resistance to certain antibiotics, such as amikacin and piperacillin-tazobactam, seem encouraging since these antibiotics will continue to be effective for the long-term treatment of *P. aeruginosa* infections. Hence, hospitals should establish guidelines and modify the antibiotic policy, including rotational and stop policies, to increase the availability of antibiotics for the treatment of *P. aeruginosa*. Furthermore, the findings of this study suggest that the prominent mechanisms of resistance to fluoroquinolone for clinical strains of *P. aeruginosa* include mutations in *gyrA* and *parC* genes. The limitation of this study is the inclusion of only one general hospital in Kuwait, therefore limiting the ability to generalize the findings to all hospitals in Kuwait. In addition, some of the isolates in this study appeared resistant phenotypically, but the resistance genes were not detected by molecular methods. Therefore, whole-genome sequencing is recommended to determine the basis of resistance.

## Figures and Tables

**Table 1:** The bacterial species isolated from various types of samples.

Organism	Respiratory	Blood	Urine	Others
*Klebsiella pneumonia*	46	3	3	5
*Acinetobacter baumannii*	52	0	0	0
*Pseudomonas aeruginosa*	46	4	22	36
*Escherichia coli*	0	0	0	1

**Table 2:** The bacterial species isolated from samples obtained from wards and ICUs.

Organism	Wards Samples	ICUs Samples
*Klebsiella pneumonia*	27	30
*Acinetobacter baumannii*	15	37
*Pseudomonas aeruginosa*	90	18
*Escherichia coli*	1	0

**Table 3:** The number of resistant isolates to various antibiotics among the ward and ICU isolates.

Antibiotic Name	Number of Resistances Isolates from Wards, n(%)	Number of Resistances Isolates from ICUs’, n (%)
Piperacillin-tazobactam	6 (7.3%)	1 (5.5%)
Ceftazidime-avibactam	23 (28%)	3 (16.6%)
Ceftolozane-tazobactam	21 (25.6%)	1 (5.5%)
Aztreonam	19 (23.1%)	2 (11.1%)
Colistin	9 (10.9%)	1 (5.5%)
Tobramycin	17 (20.7%)	1 (5.5%)
Amikacin	14 (17%)	1 (5.5%)
Gentamicin	19 (23.1%)	1 (5.5%)
Ciprofloxacin	25 (30.4%)	3 (16.6%)
Meropenem	26 (31.7%)	2 (11.1%)
Imipenem	27 (32.9%)	3 (16.6%)
Ceftazidime	22 (26.8%)	3 (16.6%)

**Table 4:** Mutations detected in the *gyrA* and *parC* genes among 15 MDR *P. aeruginosa* isolates.

Isolate #	*gyrA* Position		*parC* Position	
	Point Mutation	Silent Mutation	Point Mutation	Silent Mutation
69	Thr>Ile ACC83ATC	-	Ser>Leu TCG87TTG	Ala>Ala GCG115GCT
71	Thr>Ile ACC83ATC	-	Ser>Leu TCG87TTG	Ala>Ala GCG115GCT
72	Thr>Ile ACC83ATC	-	Ser>Leu TCG87TTG	Ala>Ala GCG115GCT
77	Thr>Ile ACC83ATC	-	Ser>Leu TCG87TTG	Ala>Ala GCG115GCT
94	Thr>Ile ACC83ATC	-	Ser>Leu TCG87TTG	Ala>Ala GCG115GCT
95	Thr>Ile ACC83ATC	Arg>Arg CGT68CGA His>His CAC132CAT	Ser>Leu TCG87TTG	Ala>Ala GCG115GCT
99	Thr>Ile ACC83ATC	His>His CAC132CAT	-	Ala>Ala GCG115GCT
123	No mutation		-	Ala>Ala GCG115GCT
160	Thr>Ile ACC83ATC	Arg>Arg CGT68CGA His>His CAC132CAT	Ser>Leu TCG87TTG	Ala>Ala GCG115GCT
182	Thr>Ile ACC83ATC	-	-	Ala>Ala GCG115GCT
194	Thr>Ile ACC83ATC	-	Ser>Leu TCG87TTG	Ala>Ala GCG115GCT
196	Thr>Ile ACC83ATC	Arg>Arg CGT68CGA	Ser>Leu TCG87TTG	Ala>Ala GCG115GCT
201	Thr>Ile ACC83ATC	-	Ser>Leu TCG87TTG	Ala>Ala GCG115GCT
203	Thr>Ile ACC83ATC	His>His CAC132CAT	Ser>Leu TCG87TTG	Ala>Ala GCG115GCT
204	Thr>Ile ACC83ATC	-	Ser>Leu TCG87TTG	Ala>Ala GCG115GCT

**Table 5:** The PCR protocols and the target genes for various antibiotic groups.

Antibiotic Group	Gene	Denaturation	Annealing	Extension	References
β-lactam-β-lactamase inhibitor combinations (amoxicillin-clavulanic acid)	*bla-TEM*, *bla-SHV*, and *bla-OXA-10*	12 min at 95 °C/30 cycles	50 °C for 30 s	72 °C for 1 min	[48,49]
ESBL genes	*bla-CTX*, *bla-TEM*, *bla-SHV*, and *bla-VEB*	12 min at 95 °C/30 cycles	50 °C for 30 s	72 °C for 1 min	[48,50]
Carbapenems	*bla-IMP*, *bla-VIM*, *bla-OXA-48*, *bla-OXA-23*, *bla-NDM*	12 min at 95 °C/30 cycles	50 °C for 30 s	72 °C for 1 min	[50,51]
Aminoglycosides	*aac(6′)-Ib*, *ant(2″)-Ia*, *ant(3″)-Ia*, *aph(3′)-Ib*, and *aac(3)-Ia*	12 min at 95 °C/30 cycles	50 °C for 30 s	72 °C for 1 min	[35,36,40]
Fluoroquinolones	*qnrA*, *qnrB*, *qnrC*, *qnrD*, and *qnrS*	12 min at 95 °C/30 cycles	50 °C for 30 s	72 °C for 1 min	[40]

## Data Availability

Data are contained within the article and Supplementary Materials.

## Supplementary Materials

The following supporting information can be downloaded at: https://www.mdpi.com/article/10.3390/antibiotics14060539/s1, Table S1: List of primers used for PCRs and sequence analysis, Table S2: Summary of the results for testing *P. aeruginosa* isolates for susceptibility/resistance profile, and MDR to various antibiotics.

## Abbreviations

The following abbreviations are used in this manuscript:

AAC	Aminoglycoside acetyltransferase
AMEs	Aminoglycoside modifying enzymes
AMI	Amikacin
AMR	Antimicrobial resistance
ANT	Aminoglycoside nucleotidyl transferase
APH	Aminoglycoside phosphoryl transferase
Asn	Asparagine
Asp	Aspartic acid or aspartate
AUG	Amoxicillin-clavulanic acid
AZT	Aztreonam
C/T	Ceftolozane-tazobactam
CDC	Centers for Disease Control and Prevention
CIP	Ciprofloxacin
CLSI	Clinical Laboratory Standards Institute
COL	Colistin
CZA	Ceftazidime-avibactam
ESBL	Extended-Spectrum Beta-Lactamase
ETP	Ertapenem
FOT	Cefotaxime
GEN	Gentamicin
Gly	Glycine
ICUs	Intensive Care Units
IMI	Imipenem
MDR	Multidrug-resistant
MERO	Meropenem
MIC	Minimum inhibitory concentration
*P. aeruginosa*	*Pseudomonas aeruginosa*
P/T4	Piperacillin-tazobactam
PBP3	Penicillin-binding protein
PCR	Polymerase chain reaction
QRDR	Quinolone-resistant determinative region
RND	Resistance-nodulation-division
SXT	Trimethoprim-sulfamethoxazole
TAZ	Ceftazidime
TGC	Tigecycline
TOB	Tobramycin
WHO	World Health Organization

## References

[1] Wilson M.G., Pandey S. *Pseudomonas Aeruginosa*. StatPearls-NCBI Bookshelf. https://www.ncbi.nlm.nih.gov/books/NBK557831/.

[2] Mulcahy L.R., Isabella V.M., Lewis K. (2013). *Pseudomonas aeruginosa* biofilms in disease. Microb. Ecol..

[3] (2022). Antimicrobial Resistance Collaborators Global burden of bacterial antimicrobial resistance in 2019: A systematic analysis. Lancet.

[4] (2024). WHO Bacterial Priority Pathogens List, 2024: Bacterial Pathogens of Public Health Importance to Guide Research, Development, and Strategies to Prevent and Control Antimicrobial Resistance.

[5] Pai H., Kim J.-W., Kim J., Lee J.H., Choe K.W., Gotoh N. (2001). Carbapenem resistance Mechanisms in *Pseudomonas aeruginosa* Clinical Isolates. Antimicrob. Agents Chemother..

[6] Glen K.A., Lamont I.L. (2024). Characterization of acquired β-lactamases in *Pseudomonas aeruginosa* and quantification of their contributions to resistance. Microbiol. Spectr..

[7] Michaelis C., Grohmann E. (2023). Horizontal gene transfer of antibiotic resistance genes in biofilms. Antibiotics.

[8] Ramirez M.S., Tolmasky M.E. (2010). Aminoglycoside modifying enzymes. Drug Resist. Updates Rev. Comment. Antimicrob. Anticancer. Chemother..

[9] Collins J.A., Osheroff N. (2024). Gyrase and Topoisomerase IV: Recycling old targets for New Antibacterials to Combat Fluoroquinolone Resistance. ACS Infect. Dis..

[10] Pachori P., Gothalwal R., Gandhi P. (2019). Emergence of antibiotic resistance Pseudomonas aeruginosa in intensive care unit; a critical review. Genes. Dis..

[11] Li Y., Roberts J.A., Walker M.M., Aslan A.T., Harris P.N.A., Sime F.B. (2024). The global epidemiology of ventilator-associated pneumonia caused by multi-drug resistant Pseudomonas aeruginosa: A systematic review and meta-analysis. J. Infect. Dis..

[12] Ranke T.D., Strassle P., Harris A.D., Zhu J., Johnson J.K. (2012). Recovery of Gram-negative bacilli in stored endotracheal aspirates. J. Clin. Microbiol..

[13] Al-Orphaly M., Hadi H.A., Eltayeb F.K., Al-Hail H., Samuel B.G., Sultan A.A., Skariah S. (2021). Epidemiology of Multidrug-Resistant *Pseudomonas aeruginosa* in the Middle East and North Africa Region. mSphere.

[14] Hays J.P., Safain K.S., Almogbel M.S., Habib I., Khan M.A. (2022). Extended Spectrum- and Carbapenemase-Based Β-Lactam resistance in the Arabian Peninsula—A descriptive review of recent years. Antibiotics.

[15] Boushra M.R., Gad G.F.M., Hassuna N.A., Waly N.G.F., Ibrahem R.A. (2024). Phenotypic and genotypic assessment of fluoroquinolones and aminoglycosides resistance in Pseudomonas aeruginosa collected from Minia hospitals, Egypt during COVID-19 pandemic. BMC Infect. Dis..

[16] Arefin M.S., Mitu M.J., Mitu S.Y., Nurjahan A., Mobin M., Nahar S., Rahman M.H. (2025). Mutational alterations in the QRDR regions associated with fluoroquinolone resistance in Pseudomonas aeruginosa of clinical origin from Savar, Dhaka. PLoS ONE.

[17] Hafiz T.A., Essa E.A.B., Alharbi S.R., Alyami A.S., Alkudmani Z.S., Mubaraki M.A., Alturki N.A., Alotaibi F. (2023). Epidemiological, microbiological, and clinical characteristics of multi-resistant *Pseudomonas aeruginosa* isolates in King Fahad Medical City, Riyadh, Saudi Arabia. Trop. Med. Infect. Dis..

[18] Alali W.Q., AlFouzan W., Dhar R. (2021). Prevalence of antimicrobial resistance in Gram-negative clinical isolates from a major secondary hospital in Kuwait: A retrospective descriptive study. GERMS.

[19] Yoon E.J., Jeong S.H. (2021). Mobile carbapenemase genes in Pseudomonas aeruginosa. Front. Microbiol..

[20] Maharjan N. (2022). Pseudomonas aeruginosa isolates among clinical samples showing growth in a tertiary care centre: A descriptive cross-sectional study. J. Nep. Med. Assoc..

[21] Alshammari H.O., Somily A., Qattan M.Y., Alsubki R.A., Moussa I.M. (2023). Susceptibility pattern of multi-drug resistance Pseudomonas aeruginosa isolates from tertiary care hospital in Riyadh, KSA. J. King Saud Univ. Sci..

[22] Patel J., Javiya V., Ghatak S., Patel K. (2008). Antibiotic susceptibility patterns of *Pseudomonas aeruginosa* at a tertiary care hospital in Gujarat, India. Indian. J. Pharmacol..

[23] Alqahtani M., Tickler I.A., Deesi Z.A., AlFouzan W., Jabri A.A., Jindan R.A., Johani S.A., Alkahtani S.A., Kharusi A.A., Mokaddas E. (2021). Molecular detection of carbapenem resistance genes in rectal swabs from patients in Gulf Cooperation Council hospitals. J. Hosp. Infect..

[24] Khater E. (2022). Detection of carbapenem-resistant *Pseudomonas aeruginosa* in tertiary care hospital in Saudi Arabia. Microb. Infect. Dis..

[25] Gondal A.J., Choudhry N., Niaz A., Yasmin N. (2024). Molecular analysis of carbapenem and aminoglycoside resistance genes in carbapenem-resistant *Pseudomonas aeruginosa* clinical strains: A Challenge for Tertiary Care Hospitals. Antibiotics.

[26] Hosu M.C., Vasaikar S.D., Okuthe G.E., Apalata T. (2021). Detection of extended spectrum beta- lactamase genes in *Pseudomonas aeruginosa* isolated from patients in rural Eastern Cape Province, South Africa. Sci. Rep..

[27] Haghighi S., Goli H.R. (2022). High prevalence of blaVEB, blaGES, and blaPER genes in beta- lactam resistant clinical isolates of Pseudomonas aeruginosa. AIMS Microbiol..

[28] Jalal N.A., Hariri S.H., Momenah A.M., Khan S., Bantun F. (2023). Molecular detection of blaPER-1, blaVEB-1, and blaPSE-1 βlactamase genes from P. aeruginosa Severe Urogenital UTI Infection. Fish. Sci..

[29] Mohammedkheir M.I.A., Gaafar E.M., AbdAlla E.G.E. (2024). Assessment of Bla TEM, Bla SHV, and Bla CTX-M genes of antibiotic resistance in Gram-negative bacilli causing urinary tract infections in Khartoum State: A cross-sectional study. BMC Infect. Dis..

[30] Bahrami M., Mmohammadi-Sichani M., Karbasizadeh V. (2018). Prevalence of SHV, TEM, CTX-M and OXA-48 β-Lactamase Genes in Clinical Isolates of *Pseudomonas aeruginosa* in Bandar-Abbas, Iran. Avicenna J. Clin. Microbiol. Infect..

[31] Marwa M.A., Atef S., Mai M. (2015). OXA-10 and GES-1 extended-spectrum beta-lactamases play a major role in causing antibiotic resistance of *Pseudomonas aeruginosa* isolated from nosocomial infections in Ismailia, Egypt. Egypt. J. Med. Microbiol..

[32] Hashemi A.B., Moghaddam M.N., Forghanifard M.M., Yousefi E. (2021). Detection of blaOXA-10 and blaOXA-48 Genes in *Pseudomonas aeruginosa* Clinical Isolates by Multiplex PCR. J. Med. Microbiol. Infect. Dis..

[33] Thabit A.K., Alghamdi A.M., Miaji M.Y., Alharbi F.S., Jawah A.F., Alturki F., Hosin N., Bazuqamah M., Almutairi M.S., Alhamed H. (2024). Antibiotic susceptibility of *Pseudomonas aeruginosa* in Saudi Arabia: A national antimicrobial resistance surveillance study. Front. Public. Health.

[34] Atassi G., Medernach R., Scheetz M., Nozick S., Rhodes N.J., Murphy-Belcaster M., Hauser A.R. (2023). Genomics of aminoglycoside resistance in Pseudomonas aeruginosa bloodstream infections at a United States Academic Hospital. Microbiol. Spectr..

[35] Azimi L., Armin S., Kafil H.S., Abdollahi N., Ghazvini K., Hasanzadeh S., Zahedani S.S., Tabatabaei S.R., Fallah F. (2022). Evaluation of phenotypic and genotypic patterns of aminoglycoside resistance in the Gram-negative bacteria isolates collected from pediatric and general hospitals. Mol. Cell Pediatr..

[36] Ahmadian L., Bazgir Z.N., Ahanjan M., Valadan R., Goli H.R. (2021). Role of Aminoglycoside- modifying enzymes (AMEs) in resistance to aminoglycosides among clinical isolates of *Pseudomonas aeruginosa* in the north of Iran. BioMed Res. Int..

[37] Abdelrahim S.S., Hassuna N.A., Waly N.G., Kotb D.N., Abdelhamid H., Zaki S. (2024). Coexistence of plasmid-mediated quinolone resistance (PMQR) and extended-spectrum beta-lactamase (ESBL) genes among clinical Pseudomonas aeruginosa isolates in Egypt. BMC Microbiol..

[38] Alfouzan W., Dhar R., Nicolau D.P. (2018). In vitro activity of newer and conventional antimicrobial agents, including fosfomycin and colistin, against selected Gram-negative bacilli in Kuwait. Pathogens.

[39] Benwan K.A., Jamal W. (2022). Etiology and antibiotic susceptibility Patterns of urinary tract infections in children in a general hospital in Kuwait: A 5-ear retrospective study. Med. Princ. Pract..

[40] Saki M., Sheikh A.F., Seyed-Mohammadi S., Dezfuli A.A.Z., Shahin M., Tabasi M., Veisi H., Keshavarzi R., Khani P. (2022). Occurrence of plasmid-mediated quinolone resistance genes in *Pseudomonas aeruginosa* strains isolated from clinical specimens in southwest Iran: A multicentral study. Sci. Rep..

[41] Shariati A., Arshadi M., Khosrojerdi M.A., Abedinzadeh M., Ganjalishahi M., Maleki A., Heidary M., Khoshnood S. (2022). The resistance mechanisms of bacteria against ciprofloxacin and new approaches for enhancing the efficacy of this antibiotic. Front. Public. Health.

[42] El-Badawy M.F., Alrobaian M.M., Shohayeb M.M., Abdelwahab S.F. (2019). Investigation of six plasmid-mediated quinolone resistance genes among clinical isolates of pseudomonas: A genotypic study in Saudi Arabia. Infect. Drug Resist..

[43] Shanan R., Yousef N., Balid M.E., Tahan Z.S. (2025). Prevalence of Plasmid-Mediated Fluoroquinolone Resistance Genes in Pseudomonas aeruginosa Among Patients at Aleppo University Hospital, Syria. J. Clin. Lab. Anal..

[44] Al-Marjani M.F. (2014). Presence of qnr gene in environmental and clinical *Pseudomonas aeruginosa* isolates in Baghdad. Int. J. Curr. Microbiol. Appl. Sci..

[45] Akhlaghi F., Nikokar I., Mojtahedi A., Mobin M., Atrkar Roshan Z., Karampour M. (2024). Molecular detection of mutations in gyrA, gyrB, parC, and parE genes in the quinolone resistance determining region among Pseudomonas aeruginosa isolated from burn wound infection. Iran. J. Med. Microbiol..

[46] Pang Z., Raudonis R., Glick B.R., Lin T.-J., Cheng Z. (2018). Antibiotic resistance in *Pseudomonas aeruginosa*: Mechanisms and alternative therapeutic strategies. Biotechnol. Adv..

[47] Alajmi R.Z., Alfouzan W.A., Mustafa A.S. (2023). The prevalence of multidrug-resistant Enterobacteriaceae among neonates in Kuwait. Diagnostics.

[48] Neyestanaki D.K., Mirsalehian A., Rezagholizadeh F., Jabalameli F., Taherikalani M., Emaneini M. (2014). Determination of extended spectrum beta-lactamases, metallo-beta-lactamases and AmpC-beta- lactamases among carbapenem resistant *Pseudomonas aeruginosa* isolated from burn patients. Burns.

[49] Bert F. (2002). Identification of PSE and OXA beta-lactamase genes in *Pseudomonas aeruginosa* using PCR- restriction fragment length polymorphism. J. Antimicrob. Chemother..

[50] Cho H.H., Kwon G.C., Kim S., Koo S.H. (2015). Distribution of pseudomonas-derived cephalosporinase and metallo- β -lactamases in carbapenem-resistant *Pseudomonas aeruginosa* isolates from Korea. J. Microbiol. Biotechnol..

[51] Khosravi A.D., Mihani F. (2007). Detection of metallo-β-lactamase–producing *Pseudomonas aeruginosa* strains isolated from burn patients in Ahwaz, Iran. Diagn. Microbiol. Infect. Dis..

[52] Michalska A.D., Sacha P.T., Ojdana D., Wieczorek A., Tryniszewska E. (2014). Prevalence of resistance to aminoglycosides and fluoroquinolones among *Pseudomonas aeruginosa* strains in a University Hospital in Northeastern Poland. Braz. J. Microbiol..

[53] Farahi R.M., Ali A.A., Gharavi S. (2018). Characterization of *gyrA* and *parC* mutations in ciprofloxacin-resistant *Pseudomonas aeruginosa* isolates from Tehran hospitals in Iran. Iran. J. Microbiol..

