# Supplementary material for: Antibiotics Resistance Profile of Clinical Isolates of Pseudomonas aeruginosa Obtained from Farwaniya Hospital in Kuwait Using Phenotypic and Molecular Methods
Source: Antibiotics (Basel). 2025 May 24;14(6):539. doi: 10.3390/antibiotics14060539 (PMC12189386; doi:10.3390/antibiotics14060539)
Supplement: Supplementary file 1 [file antibiotics-14-00539-s001.zip › antibiotics-3612475-supplementary.pdf]

Table S1. List of primers used for PCRs and sequence analysis.

Antibiotic	Target gene	Sequence	Size of amplified DNA (bp)	Reference
1st , 2nd , 3rd generation cephalosporin	*bla-TEM*	F-CTTCCTGTTTTTGCTCACC<br>R-AGCAATAAACCAGCCAGC	636	[48]
	bla-SHV	F-TCAGCGAAAAACACCTTG<br>R-TCCCGCAGATAAATCACC	472	[48]
	bla-CTX	F - GCGATGGGCAGTACCAGTAA<br>R - TTACCCAGCGTCAGATTCCG	392	[48]
Beta lactamase inhibitors	*bla-TEM*	F-CTTCCTGTTTTTGCTCACC<br>R-AGCAATAAACCAGCCAGC	636	[48]
	bla-SHV	F-TCAGCGAAAAACACCTTG<br>R-TCCCGCAGATAAATCACC	472	[48]
	bla-OXA-10	F-TTTCGAGTACGGCATTAGCT<br>R-GACACCAGGATTTGACTCAG	276	[49]
	bla-VEB	F-CATTTCCCGATGCAAAGCGT<br>R-CGAAGTTTCTTTGGACTCTG	648	[50]
Carbapenem	*bla-IMP*	F-GAAGGCGTTTATGTTCATAC<br>R-GTATGTTTCAAGAGTGATGC	587	[51]
	bla-VIM	F-GATGGTGTTTGGTCGCATA<br>R-CGAATGCGCAGCACCAG	390	[51]

	bla-OXA-48	F - GCGTGGTTAAGGATGAACAC<br>R - CATCAAGTTCAACCCAACCG	438	[51]
	bla-NDM	F-GGTTTGGCGATCTGGTTTTC<br>R-CGGAATGGCTCATCACGATC	621	[51]
	bla-OXA-23	F-GATCGGATTGGAGAACCAGA<br>R-ATTTCTGACCGCATTTCCAT	501	[51]
Fluoroquinolones	*qnr A*	F-ATTTCTCACGCCAGGATTTG<br>R-GATCGGCAAAGGTTAGGTCA	516	[40]
	qnr B	F-GATCGTGAAAGCCAGAAAGG<br>R-ATGAGCAACGATGCCTGGTA	476	[40]
	qnr C	F-GGGTTGTACATTTATTGAATCG<br>R-CACCTACCCATTTATTTTCA	307	[40]
	qnr D	F-CGAGATCAATTTACGGGGAATA<br>R-AACAAGCTGAAGCGCCTG	582	[40]
	qnr S	F- GCAAGTTCATTGAACAGGGT<br>R-TCTAAACCGTCGAGTTCGGCG	428	[40]
	gyr A	F-GTGTGCTTTATGCCATGAG<br>R- GGTTTCCTTTTCCAGGTC	287	[53]

	par C	F- CATCGTCTACGCCATGAG<br>R- AGCAGCACCTCGGAATAG	267	[53]
Aminoglycosides	*aac (6')-Ib*	F-TTGCGATGCTCTATGAGTGGCTA<br>R-CTCGAATGCCTGGCGTGTTT	482	[35]
	ant(3'')-Ia	*F- TGTAGAAGTCACCATTGTTG*<br>*R-TCAGCAAGATAGCCAGAT*	152	[36]
	ant(2'')-Ia	F-GACACAACGCAGGTCACATT<br>R-CGCAAGACCTCAACCTTTTC	500	[52]
	aph(3')-I b	F-CTTGGTGATAACGGCAATTTC<br>R-CCAATCGCAGATAGAAGGCAA	548	[52]
	aac(3)-Ia	F-GGCTCAAGTATGGGCATCAT<br>R-TCACCGTAATCTGCTTGCAC	389	[52]

*F: Forward, R: Reverse.

Table S2. Summary of the results for testing *P. aeruginosa* isolates for susceptibility/resistance profile, and MDR to various antibiotics.

Isolate #	Susceptible antibiotics	Intermediate antibiotics	Resistance antibiotics	MDR	Antibiotics resistance genes
69	None	Polymyxin	Beta lactams<br>Aminoglycosides<br>Fluoroquinolones<br>P/T4 , C/T, CZA	Yes	*blaVEB*<br>*blaNDM*<br>*qnr S*
70	Cephalosporins<br>Imipenem<br>Aztreonam<br>P/T4 , C/T, CZA	Polymyxin	Meropenem	No	
71	None	P/T4<br>Polymyxin	Beta lactams<br>Aminoglycosides<br>Fluoroquinolones<br>C/T, CZA	Yes	*blaVEB*<br>*blaNDM*<br>*qnr S*
72	None	P/T4<br>Polymyxin	Beta lactams<br>Aminoglycosides	Yes	*blaVIM*

			Fluoroquinolones<br>C/T, CZA		
73	Aminoglycosides<br>Fluroquinolones<br>C/T, P/T4	Polymyxin<br>Monobactam<br>Cephalosporins<br>Imipenem	CZA<br>Meropenem	No	
74	Carbapenems<br>Monobactam<br>P/T4	Polymyxin<br>Cephalosporins<br>Aminoglycosides	C/T<br>Fluroquinolones	No	
75	Beta lactams<br>Aminoglycosides<br>Fluroquinolones<br>C/T, CZA, P/T4	Polymyxin	None	No	
76	Beta lactams<br>Aminoglycosides<br>Fluroquinolones<br>C/T, CZA, P/T4	Polymyxin	None	No	
77	None	Polymyxin	Beta lactams	Yes	*aac (6')-Ib*<br>*qnr S*

			Aminoglycosides<br>Fluroquinolones<br>C/T, CZA, P/T4		
78	Beta lactams<br>Aminoglycosides<br>Fluroquinolones<br>C/T, CZA, P/T4	Polymyxin	None	No	
79	Aminoglycosides<br>Fluroquinolones<br>C/T	Polymyxin<br>Beta lactams<br>P/T4	CZA	No	
94	None	Polymyxin<br>P/T4	Beta lactams<br>Aminoglycosides<br>Fluroquinolones<br>C/T, CZA	Yes	*blaVEB*<br>*blaOXA-10*<br>*ant(3'')-Ia*<br>*qnr S*
95	None	Polymyxin<br>P/T4	Beta lactams<br>Aminoglycosides<br>Fluroquinolones	Yes	*blaVIM*<br>*aph(3')-Ib*<br>*qnr S*

			C/T, CZA		
96	Beta lactams<br><br>Aminoglycosides<br><br>Fluroquinolones<br><br>C/T, CZA,P/T4	Polymyxin	None	No	
97	Beta lactams<br><br>Aminoglycosides<br><br>Fluroquinolones<br><br>C/T, CZA,P/T4	Polymyxin	None	No	
98	Beta lactams<br><br>Aminoglycosides<br><br>Fluroquinolones<br><br>C/T, CZA, P/T4	Polymyxin	None	No	
99	C/T	Polymyxin<br><br>Monobactam	Cephalosporins<br><br>Carbapenems<br><br>Aminoglycosides<br><br>Fluoroquinolone<br>s<br><br>CZA	Yes	*qnr S*

100	Monobactam<br>Cephalosporins<br>Imipenem<br>Aminoglycosides<br>C/T,P/T4	Polymyxin	Meropenem<br>Fluoroquinolones<br>CZA	yes	Not tested
101	Beta lactams<br>Aminoglycosides<br>Fluroquinolones<br>C/T, CZA,P/T4	Polymyxin	None	No	
102	Beta lactams<br>Aminoglycosides<br>C/T, CZA, P/T4	Polymyxin<br>Fluoroquinolones	None	No	
103	Beta lactams<br>Aminoglycosides<br>Fluroquinolones	Polymyxin	None	No	

	C/T, CZA, P/T4				
104	Beta lactams<br>Aminoglycosides<br>Fluroquinolones<br>C/T, CZA, P/T4	Polymyxin	None	No	
105	Beta lactams<br>Aminoglycosides<br>Fluroquinolones<br>C/T, CZA, P/T4	Polymyxin	None	No	
106	Beta lactams<br>Aminoglycosides<br>Fluroquinolones<br>C/T, CZA, P/T4	Polymyxin	None	No	
108	Aminoglycosides<br>Fluoroquinolones	Polymyxin<br>Cephalosporins<br>C/T, P/T4	Carbapenems<br>Monobactam<br>CZA	yes	Not tested

109	Cephalosporins<br><br>Monobactam<br><br>Amikacin	Polymyxin<br><br>Carbapenems<br><br>Tobramycin, Gentamicin<br><br>P/T4	Fluoroquinolones<br><br>C/T, CZA	No	
111	Carbapenems<br><br>Monobactam<br><br>Aminoglycosides<br><br>Fluoroquinolones<br><br>C/T, CZA	Polymyxin<br><br>Cephalosporins<br><br>P/T4	None	No	
113	Beta lactams<br><br>Aminoglycosides<br><br>Fluoroquinolones<br><br>C/T, CZA, P/T4	None	Polymyxin	No	
114	Cephalosporins<br><br>Monobactam	Polymyxin<br><br>Meropenem	Imipenem	No	

	Aminoglycosides<br><br>Fluoroquinolone<br>s<br><br>C/T, CZA, P/T4				
115	Beta lactams<br><br>Aminoglycosides<br><br>Fluoroquinolone<br>s<br><br>C/T, CZA	Polymyxin	P/T4	No	
116	Beta lactams<br><br>Aminoglycosides<br><br>Fluoroquinolone<br>s<br><br>C/T, CZA, P/T4	Polymyxin	None	No	
117	Beta lactams<br><br>Aminoglycosides<br><br>Fluoroquinolone<br>s<br><br>C/T, CZA, P/T4	Polymyxin<br><br>Fluoroquinolone<br>s	None	No	
118	Beta lactams<br><br>Aminoglycosides	Polymyxin	None	No	

	Fluoroquinolone<br>s<br><br>C/T, CZA, P/T4				
119	Meropenem<br><br>CZA	Polymyxin<br><br>Cephalosporins<br><br>Aminoglycosides<br><br>Imipenem<br><br>Monobactam<br><br>P/T4	C/T<br><br>Fluoroquinolone<br>s	No	
120	Imipenem<br><br>Aminoglycosides<br><br>Fluoroquinolone<br>s<br><br>C/T	Polymyxin<br><br>Cephalosporins<br><br>Meropenem<br><br>Monobactam<br><br>P/T4	CZA	No	
121	Beta lactams<br><br>Aminoglycosides<br><br>Fluoroquinolone<br>s<br><br>C/T, CZA, P/T4	Polymyxin	None	No	
122	Beta lactams	Polymyxin	None	No	

	Aminoglycosides<br><br>Fluoroquinolone<br>s<br><br>C/T, CZA, P/T4				
123	Monobactam<br><br>P/T4	Polymyxin	Aminoglycosides<br><br>Fluoroquinolone<br>s<br><br>Cephalosporins<br><br>Carbapenems<br><br>C/T, CZA	Yes	*qnr S*
124	Beta lactams<br><br>Aminoglycosides<br><br>Fluoroquinolone<br>s<br><br>C/T, CZA, P/T4	Polymyxin	None	No	
125	Beta lactams<br><br>Aminoglycosides<br><br>Fluoroquinolone<br>s<br><br>C/T, CZA, P/T4	Polymyxin	None	No	
126	Meropenem<br><br>P/T4, CZA	Polymyxin<br><br>Cephalosporins	Monobactam<br><br>Fluoroquinolone<br>s	No	

		Imipenem<br>Aminoglycosides<br>C/T			
127	Cephalosporins<br>Carbapenems<br>Aminoglycosides<br>Fluoroquinolone<br>s<br>C/T, CZA, P/T4	Polymyxin<br>Monobactam	None	No	
128	Cephalosporins<br>Meropenem<br>Monobactam<br>Aminoglycosides<br>Fluoroquinolone<br>s<br>C/T, CZA, P/T4	Polymyxin<br>Imipenem	None	No	
129	Beta lactams<br>Aminoglycosides<br>Fluoroquinolone<br>s<br>C/T, CZA, P/T4	Polymyxin	None	No	

152	Cephalosporins<br>Meropenem<br>Aminoglycosides<br>Fluoroquinolones<br>C/T, CZA, P/T4	Polymyxin<br>Monobactam	Imipenem	No	
153	Beta lactams<br>Aminoglycosides<br>Fluoroquinolones<br>C/T, CZA ,P/T4	Polymyxin	None	No	
154	Beta lactams<br>Aminoglycosides<br>Fluoroquinolones<br>C/T, CZA, P/T4	Polymyxin	None	No	
155	Meropenem<br>Amikacin, Tobramycin<br>Fluoroquinolones<br>CZA	Cephalosporins<br>Gentamicin<br>C/T, P/T4	Polymyxin<br>Imipenem<br>Monobactam	yes	Not tested

156	Cephalosporins<br>Imipenem<br>Aminoglycosides<br>Fluoroquinolones<br>C/T	Polymyxin	Meropenem<br>CZA, P/T4	yes	Not tested
157	Beta lactams<br>Aminoglycosides<br>Fluoroquinolones<br>C/T, CZA, P/T4	Polymyxin	None	No	
158	Beta lactams<br>Aminoglycosides<br>Fluoroquinolones<br>C/T, CZA, P/T4	Polymyxin	None	No	
159	Imipenem<br>Aminoglycosides<br>Fluroquinolones<br>C/T	Polymyxin<br>Cephalosporins<br>P/T4	Meropenem<br>Monobactam<br>CZA	yes	Not tested
160	Monobactam	Polymyxin	Cephalosporins	Yes	*blaVIM*

		P/T4	Carbapenems<br><br>Aminoglycosides<br><br>Fluoroquinolones<br><br>C/T, CZA		
162	Beta lactams<br><br>Aminoglycosides<br><br>Fluoroquinolones<br><br>C/T, CZA, P/T4	Polymyxin	None	No	
163	Beta lactams<br><br>Aminoglycosides<br><br>Fluoroquinolones<br><br>C/T, CZA, P/T4	Polymyxin	None	No	
164	Beta lactams<br><br>Aminoglycosides<br><br>Fluoroquinolones<br><br>C/T, CZA, P/T4	Polymyxin	None	No	
165	Imipenem<br><br>Monobactam	Polymyxin<br><br>Cephalosporins	Meropenem<br><br>CZA	No	

	Aminoglycosides<br><br>Fluoroquinolones<br><br>C/T	P/T4			
166	Aminoglycosides<br><br>Fluoroquinolones<br><br>C/T, P/T4	Polymyxin<br><br>Cephalosporins<br><br>Imipenem	Meropenem<br><br>Monobactam<br><br>CZA	yes	Not tested
168	Carbapenems<br><br>Monobactam<br><br>CZA, P/T4	Polymyxin	Cephalosporins<br><br>Aminoglycosides<br><br>Fluoroquinolones<br><br>C/T	yes	Not tested
169	Tobramycin<br><br>Meropenem<br><br>Monobactam<br><br>Aminoglycosides<br><br>C/T, CZA, P/T4	Amikacin	Gentamicin<br><br>Imipenem<br><br>Cephalosporins<br><br>Fluoroquinolones<br><br>Colistin	yes	Not tested

170	Beta lactams<br><br>Aminoglycosides<br><br>Fluoroquinolones<br><br>C/T, CZA, P/T4	Polymyxin	None	No	
171	Beta lactams<br><br>Aminoglycosides<br><br>Fluoroquinolones<br><br>C/T, CZA, P/T4	Polymyxin	None	No	
172	Beta lactams<br><br>Aminoglycosides<br><br>Fluoroquinolones<br><br>C/T, CZA, P/T4	Polymyxin	None	No	
173	Carbapenems<br><br>Aminoglycosides<br><br>Fluoroquinolones<br><br>CZA	Polymyxin	Cephalosporins<br><br>Monobactam<br><br>C/T, P/T4	Yes	Not tested

174	Carbapenems<br>Aminoglycosides<br>Fluoroquinolones<br>CZA	Polymyxin	Cephalosporins<br>Monobactam<br>C/T, P/T4	Yes	Not tested
182	None	Polymyxin<br>P/T4	Beta lactams<br>Aminoglycosides<br>Fluoroquinolones<br>C/T, CZA	Yes	*blaIMP*<br>*aac (6')-Ib*<br>*qnr S*
183	Tobramycin	Polymyxin<br>Monobactam<br>Imipenem<br>P/T4	Cephalosporins<br>Meropenem<br>Fluoroquinolones<br>Gentamycin, Amikacin<br>C/T, CZA	Yes	Not tested
184	Beta lactams<br>Aminoglycosides	None	Polymyxin	No	

	Fluoroquinolones<br>C/T , CZA, P/T4				
187	Tobramycin<br>Fluoroquinolones<br>CZA, P/T4	Amikacin	Cephalosporins<br>Imipenem<br>Gentamycin<br>Polymyxin<br>C/T	Yes	Not tested
188	Beta lactams<br>Amikacin, Tobramycin<br>C/T, CZA, P/T4	Polymyxin<br>Gentamicin	Fluoroquinolones	No	
189	Beta lactams<br>Aminoglycosides<br>Fluoroquinolones<br>C/T, CZA, P/T4	Polymyxin	None	No	
190	Beta lactams<br>Aminoglycosides<br>Fluoroquinolones	Polymyxin	None	No	

	C/T, CZA, P/T4				
191	Beta lactams<br>Aminoglycosides<br>Fluoroquinolones<br>C/T, CZA, P/T4	Polymyxin	None	No	
192	Meropenem<br>Monobactam<br>Aminoglycosides<br>C/T.CZA, P/T4	Polymyxin<br>Gentamicin	Cephalosporins<br>Imipenem<br>Fluoroquinolones	Yes	Not tested
193	Beta lactams<br>Aminoglycosides<br>Fluoroquinolones<br>C/T, CZA, P/T4	Polymyxin	None	No	
194	C/T, CZA	Polymyxin<br>Monobactam<br>P/T4	Cephalosporin<br>Carbapenem<br>Aminoglycosides<br>Fluoroquinolones	Yes	*aac (6')-Ib*<br>*qnr S*

195	Beta lactams<br>Aminoglycosides<br>Fluoroquinolones<br>C/T, CZA, P/T4	Polymyxin	None	No	
196	Monobactam	Polymyxin<br>P/T4	Cephalosporin<br>Carbapenem<br>Aminoglycosides<br>Fluoroquinolones<br>C/T, CZA	Yes	*blaVIM*<br>*qnr S*
197	Cephalosporins<br>Aminoglycosides<br>Fluoroquinolones<br>C/T, CZA	Polymyxin<br>P/T4	Carbapenems<br>Monobactam	No	
198	Cephalosporins<br>Carbapenems<br>Amikacin<br>CZA, P/T4	Polymyxin<br>Gentamicin<br>Tobramycin	Monobactam<br>Fluoroquinolones<br>C/T	Yes	Not tested

199	Meropenem<br><br>Monobactam<br><br>Tobramycin<br><br>C/T, CZA, P/T4	Amikacin	Cephalosporins<br><br>Imipenem<br><br>Gentamicin<br><br>Fluroquinolones<br><br>Polymyxin	Yes	Not tested
200	Beta lactams<br><br>Aminoglycosides<br><br>Fluoroquinolones<br><br>C/T, CZA, P/T4	Polymyxin	None	No	
201	Monobactam	Polymyxin<br><br>P/T4	Cephalosporins<br><br>Carbapenems<br><br>Aminoglycosides<br><br>Fluroquinolones<br><br>C/T, CZA	Yes	*blaNDM*<br><br>*qnr S*
202	Cephalosporins<br><br>Carbapenems<br><br>Aminoglycosides	Polymyxin<br><br>Monobactam	None	No	

	Fluoroquinolones<br>C/T, CZA, P/T4				
203	Monobactam	Polymyxin<br>P/T4	Cephalosporins<br>Carbapenems<br>Aminoglycosides<br>Fluroquinolones<br>C/T, CZA	Yes	*blaVIM*<br>*qnr S*
204	None	Polymyxin<br>Monobactam<br>P/T4	Cephalosporins<br>Carbapenems<br>Aminoglycosides<br>Fluroquinolones<br>C/T, CZA	Yes	*blaNDM*<br>*qnr S*
205	Beta lactams<br>Aminoglycosides<br>Fluoroquinolones<br>C/T, CZA, P/T4	Polymyxin	None	No	
206	Beta lactams	Polymyxin	None	No	

	Aminoglycosides<br><br>Fluoroquinolone<br>s<br><br>C/T, CZA, P/T4				
207	Beta lactams<br><br>Aminoglycosides<br><br>Fluoroquinolone<br>s<br><br>C/T, CZA, P/T4	Polymyxin	None	No	
208	Aminoglycosides<br><br>Fluroquinolones<br><br>C/T	Polymyxin<br><br>Cephalosporins<br><br>P/T4	Carbapenems<br><br>Monobactam<br><br>CZA	Yes	Not tested
209	Meropenem<br><br>Monobactam<br><br>Aminoglycosides<br><br>Fluoroquinolone<br>s<br><br>C/T, CZA	None	Cephalosporins<br><br>Imipenem<br><br>Polymyxin<br><br>P/T4	Yes	Not tested
210	Cephalosporins<br><br>Aminoglycosides	Polymyxin	Carbapenems<br><br>Monobactam	No	

	Fluoroquinolones<br>C/T,CZA, P/T4				
211	Beta lactams<br>Aminoglycosides<br>C/T,CZA,P/T4	Polymyxin	Fluoroquinolones	No	
212	Aminoglycosides<br>Fluoroquinolones<br>C/T, CZA	Meropenem	Cephalosporins<br>Imipenem<br>Monobactam<br>Polymyxin<br>P/T4	Yes	Not tested
213	Cephalosporins<br>Meropenem<br>Monobactam<br>Aminoglycosides<br>Fluoroquinolones<br>C/T, CZA, P/T4	None	Imipenem<br>Polymyxin	No	
214	Cephalosporins	Polymyxin	None	No	

	Meropenems<br><br>Monobactam<br><br>Aminoglycosides<br><br>Fluoroquinolone<br>s<br><br>C/T, CZA, P/T4	Imipenem		No	
215	Meropenems<br><br>Monobactam<br><br>Aminoglycosides<br><br>Fluoroquinolone<br>s<br><br>C/T, CZA, P/T4	Polymyxin	Cephalosporins	No	
216	Beta lactams<br><br>Aminoglycosides<br><br>Fluoroquinolone<br>s<br><br>C/T, CZA, P/T4	Polymyxin	None	No	
217	Aminoglycosides<br><br>Fluoroquinolone<br>s<br><br>C/T, CZA	Polymyxin<br><br>Cephalosporins<br><br>P/T4	Carbapenems<br><br>Monobactam	No	
218	Beta lactams	Polymyxin	None	No	

	Aminoglycosides<br><br>Fluoroquinolone<br>s<br><br>C/T, CZA, P/T4				
